# Editorial: Advances in Porous Semiconductor Research

**DOI:** 10.3389/fchem.2020.00122

**Published:** 2020-03-06

**Authors:** Thierry Djenizian, Nicolas H. Voelcker

**Affiliations:** ^1^Mines Saint-Etienne, Center of Microelectronics in Provence, Department of Flexible Electronics, Gardanne, France; ^2^Al-Farabi Kazakh National University, Center of Physical-Chemical Methods of Research and Analysis, Almaty, Kazakhstan; ^3^Department of Materials Science and Engineering, Monash University, Clayton, VIC, Australia; ^4^Melbourne Centre for Nanofabrication, Victorian Node of the Australian National Fabrication Facility, Clayton, VIC, Australia

**Keywords:** semiconductor, porous material, microfabrication, nanomaterials, nanotechnologies

Since the discovery of the luminescent properties of porous silicon by Canham (1990), the anodization process has attracted renewed interest for the fabrication of porous semiconductors. To date, this technique is widely used to design new materials with advanced physico-chemical properties for many applications in optics, microelectronics, energy, biomedicine, etc…

This Research Topic features the most important and recent exciting results related to all aspects of manufacturing, characterization, properties, and applications of porous semiconductors (Si, Ge, III-V compounds, TiO_2_ nanotubes, etc.).

This Research Topic is a collection of articles carefully selected from 200 abstracts submitted to the 2018 Porous Semiconductors Science and Technology Conference. All abstracts were peer-reviewed by a panel composed of 6 co-chairs and 16 senior researchers from the scientific advisory board. The peer-reviewed papers have been chosen based on novelty of the work, fair representation of the scientific topics, and geographical considerations. Thus, this Research Topic involves co-authors from 31 academic institutions established in 15 countries from Europe, North America, Asia, and Australia.

The topical areas cover electrochemical and metal-assisted chemical etching, surface chemistry and functionalization, pore filling and nanoparticle decoration, novel nanostructures and microfabrication techniques, micro photonics and luminescence, micro systems and electronics, biomedical applications including diagnostics, bioimaging and drug delivery, energy harvesting, storage and conversion, sensors, as well as emerging applications.

We encourage readers to explore the review by Koshida and Nakamura who gives a broad overview of the emerging functions of nanostructured porous silicon for physical applications. There are also several examples of original articles covering the fabrication (Bastide et al.; Kolasinski et al.) and use of advanced properties of porous silicon for emerging applications like magnetic and electronic features (Granitzer et al.; Lepesant et al.), optical properties (Gonchar et al.), biosensing (Gongalsky et al.; Rajeev et al.; Segal et al.; Cozzi et al.), energy storage and harvesting (Djenizian et al.).

## Author Contributions

All authors listed have made a substantial, direct and intellectual contribution to the work, and approved it for publication.

### Conflict of Interest

The authors declare that the research was conducted in the absence of any commercial or financial relationships that could be construed as a potential conflict of interest.
